# Condition of the Ovaries and Uterus, Observed in a Young Woman Assassinated Shortly after Menstruation

**Published:** 1850-08

**Authors:** 


*Condition of the Ovaries and Uterus, observed in a young woman assassinated shortly after Menstruation.*— The researches of **MM. Pouchet, Bischoff**, and others, have placed beyond a doubt the spontaneous detachment of ovula during menstruation. The following case, recorded by **Dr. Janzer**, in the Medicinische Annalen, vol. xiii. part 4, is an additional proof; it moreover illustrates the changes which the mucous membrane of the uterus undergoes during the menstrual period.

Case.—The young girl who was the subject of the observation had menstruated four days before being murdered. She had never been pregnant. The autopsy was made sixteen hours after death. The surface of the left ovary presented a deep red spot, surrounded by finely injected vessels. This spot was formed by a small globular mass, imbedded in the ovary, and of an intense red through its whole thickness. The mass in question was separated from the tissue of the ovary by a thin yellow envelop, and was composed of fibres like those of areolar tissue, arranged in superimposed layers. The yellow envelop was formed by the same kind of fibres, among which there was a pretty considerable quantity of fat, not contained in cells. Near this body, there was seen a small yellow, spherical, modulated mass, composed of areolar tissue and fat. The right ovary contained two yellow bodies. The Fallopian tubes, which did not embrace the ovaries, were tumefied in the upper thirds. On slight pressure, a white matter issued from them, resembling pus, and entirely composed of round epithelial cells, some of which were furnished with vibratile cilia. No ovule, nor any traces of spermatozoa were found.

The uterine mucous membrane, between the body and the neck, was much swollen. In the uterus itself, it formed a velvety membrane, glossy and brilliant, easily detached with the handle of the scalpel, and presenting a fine network of vessels. This mucous membrane was evidently thickened; it was composed of the uterine glands, ranged perpendicularly alongside each other, and fitted with cylinder epithelium, not ciliated. The structure between the uterine glands was composed of a network of delicate fibres, of some nucleated cellular fibres, and of amorphous tissue. The surface of the uterus was covered with a thin layer of mucous, and lined with cylindrical epithelium, without cilia. The orifices of the Fallopian tubes were open. The vaginal mucous membrane was pale, but was only covered with a thin layer of mucous, containing epithelial cells.

It results from this observation that the mucous membrane of the uterus presents, during menstruation, characters analogous to those which exist during gestation; such as the hypertrophy of the uterine follicles, and the disappearance of vibratile cilia.—*Ibid*, from *Gazette Médicale de Paris*, 23d March, 1850.

